# B-cell subset changes and clinical predictors of telitacicept response in SLE patients with antiphospholipid antibody positivity: a prospective observational cohort study

**DOI:** 10.3389/fimmu.2026.1806712

**Published:** 2026-04-23

**Authors:** Yadi Sun, Xu Wang, Yueying Wang, Ping An, Dazhen Guo, Qian Xing

**Affiliations:** 1 Department of Rheumatology and Immunology, Qingdao Municipal Hospital, Qingdao, Shandong, China; 2 Department of Dermatology, Qingdao Municipal Hospital, Qingdao, Shandong, China; 3 Department of Rheumatology and Immunology, The Second People’s Hospital of Liaocheng, Liaocheng, Shandong, China; 4 School of Basic Medical Sciences, Southern Medical University, Guangzhou, Guangdong, China

**Keywords:** antiphospholipid antibody positivity, antiphospholipid syndrome, B cell subsets, predictor, systemic lupus erythematosus, telitacicept

## Abstract

**Objective:**

To characterize the dynamic changes in B-cell subsets in Systemic lupus erythematosus (SLE) patients with antiphospholipid antibody (aPL) positivity treated with telitacicept and to identify baseline predictors of treatment response.

**Methods:**

Twenty patients with active aPL-positive SLE receiving telitacicept in addition to standard therapy were enrolled, and 20 healthy individuals served as controls. Peripheral blood B-cell subsets and clinical parameters were assessed at baseline, week 12 and week 24. B cell subsets were analyzed using flow cytometry. Multivariable logistic regression was used to identify baseline predictors of SRI-4 response at week 24.

**Results:**

At baseline, the proportions of double-negative (DN) B cells and plasmablasts among total B cells were significantly higher in patients with aPL-positive SLE than in healthy controls (both *p* < 0.001). During the treatment of telitacicept, DN B-cell proportions decreased significantly by week 12 and continued to decline over 24 weeks, while plasmablast proportions decreased significantly by week 24; absolute counts of both subsets did not change significantly but showed a downward trend. Levels of BAFF, APRIL, IFN-γ, IL-21 and IL-10 were significantly higher in patients with aPL-positive SLE than in healthy controls and decreased after 24 weeks of telitacicept treatment. At week 24, 65% (13/20) of patients achieved an SRI-4 response, with significant reductions in SLEDAI-2K scores, IgG, anti-double-stranded DNA (anti-dsDNA), and 24-h urine protein, along with increased complement C3 levels. Levels of aCL IgG (*p* < 0.001), anti-β_2_GPI IgG (*p* = 0.023), and LAC (*p* = 0.025) were all reduced. Multivariable analysis showed that higher baseline IgG [OR = 1.95, 95% CI 1.05–3.63, *P* = 0.035] and anti-β2GPI IgG levels [OR = 1.06, 95% CI 1.01–1.12, *P* = 0.048] were independently associated with SRI-4 response at week 24.

**Conclusion:**

Telitacicept may represent a safe and effective therapeutic option for patients with aPL-positive SLE, potentially exerting its therapeutic effects by influencing B-cell subsets, particularly DN B cells and plasmablasts. Elevated baseline IgG and anti-β2GPI IgG levels may predict a favorable response to telitacicept, and help identify patients who are more likely to benefit from this treatment.

## 1 Introduction

SLE is a chronic autoimmune disease in which the immune system attacks healthy tissues and cells (1, 2). aPL are the characteristic serological marker of antiphospholipid syndrome (APS), a systemic autoimmune disorder characterized by recurrent vascular thrombosis and pregnancy morbidity (3). Notably, aPL are also detected in a substantial proportion of patients with SLE, and their presence is linked with thrombotic risk and adverse pregnancy outcomes (4).

Increasing evidence suggests that B cell dysregulation contributes to both SLE pathogenesis and aPL production (5–8). Although glucocorticoids and immunosuppressive agents remain the cornerstone of SLE treatment and have demonstrated clear clinical benefits, their lack of long-term efficacy and safety remains a major limitation (9). Therefore, novel therapeutic options are urgently needed. Recent research has highlighted that combining B cell-targeted therapy with conventional treatments can enhance clinical efficacy, accelerate disease control, and reduce treatment-related toxicity (10, 11). Several studies have reported that patients with APS may benefit from B cell depletion using the anti-CD20 monoclonal antibody rituximab (12). However, its clinical use is limited because of the risk of severe serum reactions (13). Moreover, its therapeutic effects in APS are less pronounced than those in other autoimmune conditions (14). Telitacicept is a novel fusion protein (15, 16) that simultaneously inhibits the binding of BLyS and A proliferation-inducing ligand (APRIL) to cyclophilin ligand interactor (TACI) to suppress plasma cell differentiation and survival as well as antibody isotype switching in marginal zone and follicular B cells to exert therapeutic effects in SLE (17). Several studies have demonstrated the efficacy and safety of telitacicept in patients with SLE (7, 18) and suggest that telitacicept may be particularly advantageous in patients with persistent aPL positivity (19). However, the characteristics of B cell subsets in patients with SLE and persistent aPL positivity remain unclear.

Therefore, this study aimed to evaluate the efficacy and safety of telitacicept in combination with standard therapy in patients with SLE with persistent aPL positivity and characterize changes in B cell subsets. By evaluating shifts in B cell populations, we sought to elucidate how telitacicept reshapes the B cell compartment and determine how these changes are associated with clinical measures of disease activity. This study may provide a foundation for predictor-guided treatment strategies and improve clinical outcomes in patients with SLE.

## 2 Methods

### 2.1 Patients and healthy controls

This was a prospective observational cohort study in which all treatment regimens were prescribed at the discretion of the patients’ supervising physicians, according to routine clinical practice. Twenty Chinese patients with SLE were enrolled from the Department of Rheumatology at Qingdao Municipal Hospital between August 2022 and February 2025. Patients were diagnosed with SLE based on the 1997 American College of Rheumatology classification criteria (20) and APS according to the 2023 ACR/EULAR Antiphospholipid Syndrome Classification Criteria (3). All patients had active disease defined as a SLE Disease Activity Index 2000 (SLEDAI-2K) score ≥ 6 with standard treatment. Eligible patients met the following conditions (1): Age: 18–70 years old (2); positive aPL detected in plasma on two or more occasions at least 12 weeks apart; and (3) stable SLE treatment regimen, with patients receiving standard treatment for at least 3 months before treatment with biological agents (telitacicept). Stable standard therapy included the following drugs administered alone or together: glucocorticoids, antimalarials, and other immunosuppressants or immunomodulators, including hydroxychloroquine, mycophenolate mofetil, cyclophosphamide, tacrolimus, and cyclosporine.

The exclusion criteria were as follows (1): history of other B cell-targeted biologic therapies within 6 months preceding telitacicept treatment (2); active hepatitis or a history of severe liver disease (3); patients with immunodeficiency, uncontrolled severe infections, and active peptic ulcers (4); pregnant women, lactating women, and patients who had planned to conceive within the previous 12 months (5); a history of allergy to human biological products (6); patients who had received live vaccines in the previous month; and (7) patients who used B cell-targeted drugs or intravenous gamma globulin (IVIG) within the preceding year. Twenty age- and sex-matched healthy Chinese individuals were enrolled as healthy controls (HC). All HCs were recruited from the physical examination center, and none had received treatment with corticosteroids or immunosuppressants. This study was approved by the Ethics Committee of Qingdao Municipal Hospital. All patients and HCs signed an informed consent form to participate in the study.

Patients with SLE who were treated with the standard treatment for 3 months continued to exhibit persistent disease activity (SLEDAI-2K ≥ 6) and were subsequently treated with telitacicept. The telitacicept group comprised 20 patients (19 females and one male) who received 160 mg telitacicept weekly, whereas the HC group included 20 age- and sex-matched individuals (19 females and one male).

### 2.2 Assessments

Criteria for disease activity and treatment response in SLE: An SLE Responder Index 4 (SRI4) response was defined as: a) a reduction in SLEDAI score of ≥ 4 points; b) no new organ manifestations with a British Isles Lupus Assessment Group score of grade A and no more than one new organ manifestation reaching grade B; and c) an increase in the physician Global Assessment not exceeding 0.3 points compared with baseline (21).

### 2.3 Sample collection and processing

#### 2.3.1 Collection of clinical specimens

Serum complement levels were measured using immunochemical methods; antinuclear antibodies were detected using immunofluorescence; and anti-double-stranded DNA antibodies, IL-6, IL-21, IL-10, B cell-activating factor (BAFF), APRIL, and Interferon-gamma (IFN-γ) were measured using ELISA kits (Wuhan Cloud-Clone Biological Co., Ltd.) according to the manufacturer’s guidelines and standard operating procedures. Serum levels of anticardiolipin antibodies (aCL) IgG and anti-β2-glycoprotein I antibodies (anti-β2GPI) IgG were measured by chemiluminescent immunoassay (CLIA) using the QUANTA Flash system (INOVA Diagnostics, San Diego, CA, USA). Positivity was defined as values ≥20.0 CU, in accordance with the manufacturer’s instructions. lupus anticoagulant (LAC) was assessed using diluted Russell’s viper venom time (dRVVT) and silica clotting time (SCT) assays. A sample was considered LAC-positive if at least one of the normalized ratios for dRVVT or SCT exceeded 1.20.

#### 2.3.2 Collection of flow cytometry specimens

Peripheral blood samples were collected from all the patients and controls at baseline (T0), 12 weeks (T12w), and 24 weeks (T24w) after treatment initiation. Fasting blood samples (5 mL) were collected from all participants into sterile EDTA anticoagulant tubes. The tubes were temporarily allowed to stand before being stored in a refrigerator at 4 °C. Following treatment with a hemolytic agent (Mindray, USA), the samples were aliquoted into multiple tubes and stained with the following antibodies: CD19-PC5.5, IgM-APC, and CD3-APC-H7 (Beckman Coulter); CD27-PC5.5 (Caprico Biotechnologies); CD38-FITC, CD24-V450, and CD45-SV538 (BioLegend); and IgD-PE-Cy7 and CD27-PE (BD). Following incubation under light-protected conditions, PBS was added, and the samples were subjected to multiple centrifugation steps. Finally, samples were analyzed using a BD FACSCanto II flow cytometer (BD Biosciences) to determine cellular expression levels and to characterize peripheral B-cell, including total B cells (CD19+), transitional B cells (CD19+CD27-CD38highIgM+CD24+), naïve B cells (CD19+CD27-IgD+), unswitched memory B cells (CD19+CD27+IgD+), memory B cells (CD19+CD27+CD38dim), switched memory B cells (CD19+CD27+CD38dimIgD-IgM-), non-immunoglobulin class-switched B lymphocytes/NCS B cells (CD19+CD27+CD38dimIgM+), plasmablasts (CD19+CD27highCD38highIgD-IgM-), and double-negative (DN) B cells (CD19+CD27-IgD-).

### 2.4 Statistical analysis

Statistical analyses were performed using SPSS 27.0 software, and figures were generated using GraphPad Prism 8. Quantitative data were expressed as the mean ± standard deviation; comparisons were performed using Student’s t-test or one-way analysis of variance followed by Tukey test. Statistical significance was set at *p* < 0.05. To identify baseline predictors of SRI-4 response at week 24, univariate logistic regression analysis was first performed. Variables with P < 0.10 in the univariate analysis were subsequently included in a multivariable logistic regression model to identify independent predictors after adjusting for potential confounding factors. Given the relatively small sample size of this exploratory study, the number of variables included in the multivariable logistic regression model was restricted to minimize the risk of model overfitting. Odds ratios (ORs) with 95% confidence intervals (CIs) were calculated. A two-sided *p* value < 0.05 was considered statistically significant.

## 3 Results

### 3.1 Baseline Characteristics of Patients with aPL-positive SLE Treated with Telitacicept Combined with Standard Therapy

In patients with aPL-positive SLE, telitacicept combined with standard therapy resulted in marked immunological effects on peripheral B cell subset distribution. In this study, 20 patients with aPL-positive SLE and 20 HCs with matched demographics (age: 42.50 ± 13.15 vs. 36.25 ± 12.95 years, p = 0.138; male/female: 1/19 vs. 1/19 in the SLE and HC groups, respectively, p = 1.0). The demographic, clinical, and immunological characteristics, as well as the medication profiles of the patients with aPL-positive SLE, are outlined in **Table 1**. Most patients were female (95%), with a mean disease duration of 7.57 ± 4.66 years. At baseline, the mean SLEDAI-2K score was 9.60 ± 2.54. Among the 20 patients, six met the diagnostic criteria for APS with thrombotic events, while the remaining 14 were classified as asymptomatic aPL carriers.

**Table 1:** Baseline demographic and clinical parameters of patients with aPL-positive SLE receiving telitacicept.

Variables	Value (n = 20)
Demographic features	
Age (years)	42.50 ± 13.15
Disease duration (years, mean ± SD)	7.57 ± 4.66
Sex (female: male)	19:1
Race: Asian n (%)	20 (100)
Organ involvement (%)	
Hematologic	75
Thrombocytopenia (< 100×10^9^/L)	25
Renal	60
Mucocutaneous	20
Serositis	25
Lungs	10
Neurologic	5
Definite APS	30
Thrombotic episodes	
Arterial thrombosis	
Ischemic stroke	3 (15)
Myocardial infarction	1 (5)
Peripheral thrombosis in leg	4 (20)
Spleen infarction	1 (5)
Venous thrombosis	
Deep venous thrombosis	4 (20)
Pulmonary embolism	2 (10)
Other venous thrombosis	2 (10)
Pregnancy complications	1 (5)
Laboratory index	
ANA ≥ 1:80 (%)	100
Anti-dsDNA serum levels (IU/mL)	47.97 ± 28.73
Decreased C3, n (%)	16 (80)
C3 serum levels (g/L)	0.566 ± 0.213
Decreased C4, n (%)	8 (40)
C4 serum levels (g/L)	0.126(0.070, 0.215)
IgG (g/L)	16.63 ± 4.30
ESR (mm/h)	31.00 (14.50, 44.50)
aPL antibody profile	
aCL (IgG)	18 (90)
anti-β2GPI (IgG)	17 (85)
LAC	11 (55)
Single aPL positivity	4 (20)
Double aPL positivity	6 (30)
Triple aPL positivity	10 (50)
Disease evaluation	
SLEDAI-2K score	9.60 ± 2.54
Baseline therapy	
Corticosteroid (prednisone equivalent)	
0~≤10 mg/day, n (%)	3 (15%)
11~≤40 mg/day, n (%)	15 (75%)
> 40 mg/day, n (%)	2 (10%)
Immunomodulant treatment	
HCQ, n (%)	17 (85)
MMF, n (%)	11 (55)
CsA, n (%)	2 (10)
CTX, n (%)	3 (15)
TAC, n (%)	5 (25)
Antiplatelet/Anticoagulant drugs	
Aspirin, n (%)	14 (70)
Warfarin, n (%)	4 (20)
Aspirin + Clopidogrel, n (%)	2 (10)

*SLE*, systemic lupus erythematosus; *SLE-DAI*, Systemic Lupus Erythematosus Disease Activity Index; *C3/C4*, Complement 3/4; *M (P25, P75)* represents the median (25th percentile, 75th percentile); *aPL*, antiphospholipid antibody; *aCL*, anticardiolipin antibody; *anti-β2GPI*, anti-β2-glycoprotein I antibody; *LAC*, lupus anticoagulant; *MMF*, mycophenolate mofetil; *HCQ*, hydroxychloroquine; *CsA*, cyclosporine A; *CTX*, cyclophosphamide; *TAC*, tacrolimus.

Among the enrolled patients, six patients had a history of thrombotic events and a total of 18 thrombotic events occurred prior to telitacicept initiation, including ischemic stroke, myocardial infarction, splenic infarctions, deep vein thrombosis (DVT), pulmonary embolism (PE), and recurrent pregnancy loss. At baseline, aCL IgG was detected in 18 patients, and anti-β_2_GPI IgG was positive in 17 patients. Furthermore, four (20%), six (30%), and ten (50%) patients had single-, double-, and triple-aPL positivity, respectively.

During follow-up, all patients were maintained on telitacicept combined with standard therapy, with corticosteroids administered to all patients. Concurrent immunosuppressive agents were administered to control lupus activity, including hydroxychloroquine (17 patients, 85%), mycophenolate mofetil (11 patients, 55%), cyclosporine A (two patients, 10%), cyclophosphamide (three patients, 15%), and tacrolimus (five patients, 25%). Among the six patients with confirmed APS, four (66.7%) received warfarin, and the remaining two (33.3%) were treated with a combination of aspirin and clopidogrel. All 14 aPL carriers received primary thromboprophylaxis with low-dose aspirin.

### 3.2 Changes in Clinical and Laboratory Parameters in Patients with aPL-Positive SLE Before and After Telitacicept Treatment

**Figure 1:**
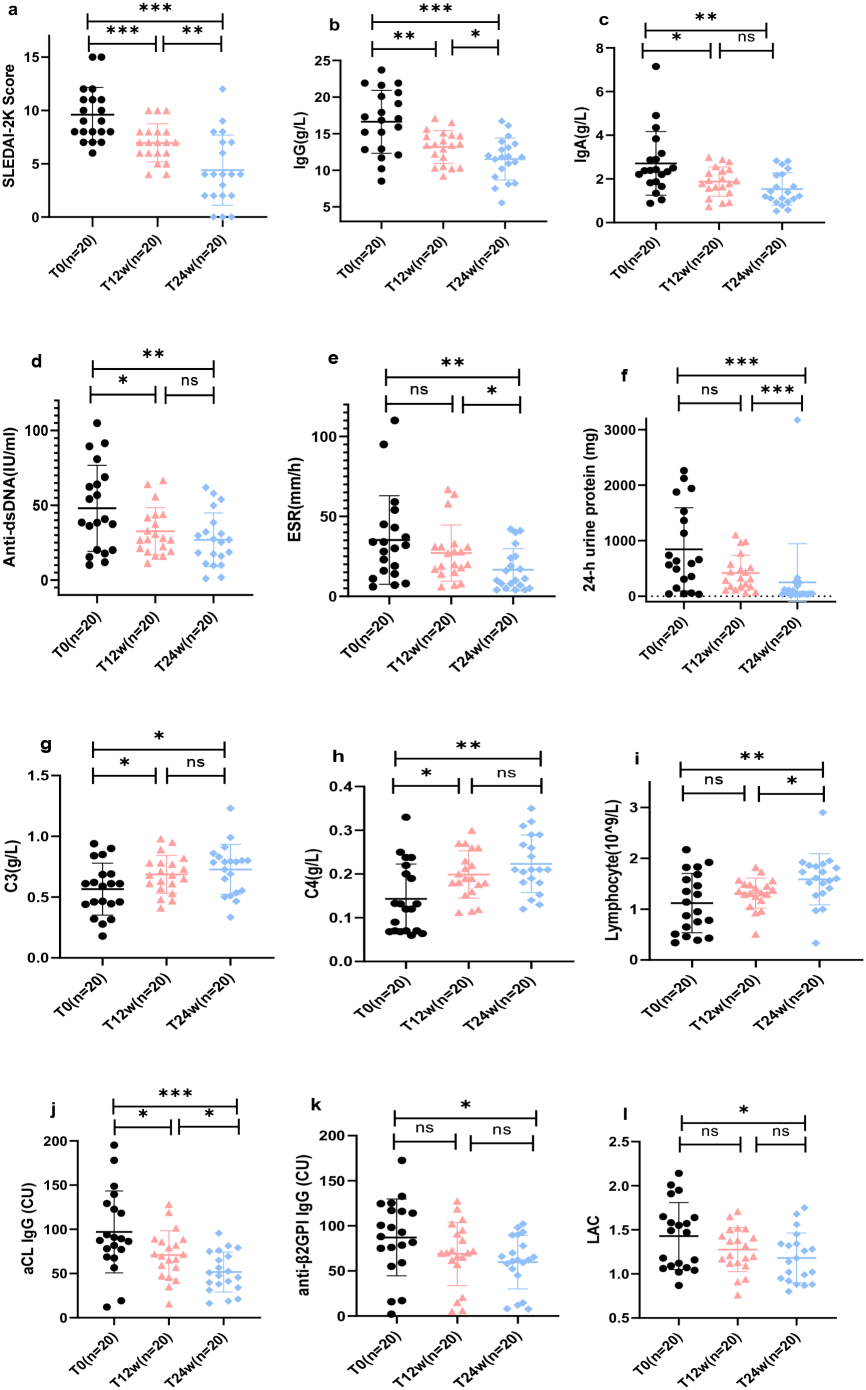
Telitacicept-associated changes in clinical markers in systemic lupus erythematosus (SLE) patients with antiphospholipid antibody (aPL) positivity. **(a)** SLEDAI-2K score; **(b)** serum IgG levels; **(c)** serum IgA levels; **(d)** anti-dsDNA antibody levels; **(e)** erythrocyte sedimentation rate (ESR); **(f)** 24-h urine protein; **(g)** complement C3 levels; **(h)** complement C4 levels; **(i)** lymphocyte counts; **(j)** anticardiolipin (aCL) IgG levels; **(k)** anti-β2 glycoprotein I (anti-β2GPI) IgG levels; **(l)** lupus anticoagulant (LAC). Data are shown for baseline (T0), week 12 (T12w), and week 24 (T24w). Abbreviations: SLE, systemic lupus erythematosus; aPL, antiphospholipid antibodies; SLEDAI-2K, Systemic Lupus Erythematosus Disease Activity Index 2000; ESR, erythrocyte sedimentation rate; aCL, anticardiolipin; anti-β2GPI, anti-β2 glycoprotein I; LAC, lupus anticoagulant. *p < 0.05; **p < 0.01; ***p < 0.001.

All 20 patients completed the 24-week follow-up, which included clinical assessments and B cell subset analysis. In patients with aPL-positive SLE, telitacicept treatment combined with standard therapy resulted in notable improvements in multiple clinical indicators. These included significant declines in SLEDAI-2K scores (T0 vs. T24w: 9.60 ± 2.54 vs. 4.40 ± 3.28, *p <* 0.001) and IgG levels (16.63 ± 4.30 vs. 11.53 ± 2.86, *p <* 0.001) following treatment (**Figure 1a, b**). Additionally, marked reductions in IgA [T0 vs. T24w: 2.39 (1.83, 3.10) vs. 1.54 ± 0.74, *p =* 0.002], anti-dsDNA (T0 vs. T24w: 47.97 ± 28.73 vs. 26.92 ± 17.94, *p =* 0.008), ESR [T0 vs. T24w: 31.00 (14.50, 44.50) vs. 10.50 (5.50, 24.75), *p =* 0.008], and 24-h urine protein [T0 vs. T24w: 612.19 (185.58, 1487.70) vs. 45.12 (36.05, 168.23)] levels were observed (**Figure 1c-f**). Moreover, increases were detected in C3 levels (T0 vs. T24w: 0.566 ± 0.213 vs. 0.727 ± 0.206, *p =* 0.020), C4 levels (T0 vs. T24w: 0.126 (0.070, 0.215) vs. 0.223 ± 0.066, *p =* 0.002), and lymphocyte counts (T0 vs. T6w: 1.12 ± 0.58 vs. 1.59 ± 0.50, *p =* 0.010) (**Figure 1g-i**). Furthermore, a significant decrease in anticardiolipin (aCL) IgG levels (T0 vs. T24w: 97.05 ± 46.32 vs. 51.74 ± 22.59, *p <* 0.001) was observed, along with reductions in anti-β2GPI IgG (T0 vs. T24w: 87.14 ± 42.57 vs. 59.71 ± 29.64, *p =* 0.023) and LAC levels (T0 vs. T24w: 1.43 ± 0.38 vs. 1.18 ± 0.28, *p =* 0.025) (**Figure 1j-l**).

### 3.3 Elevated levels of circulating DN B cells and plasmablasts in patients with aPL-positive SLE

**Figure 2:**
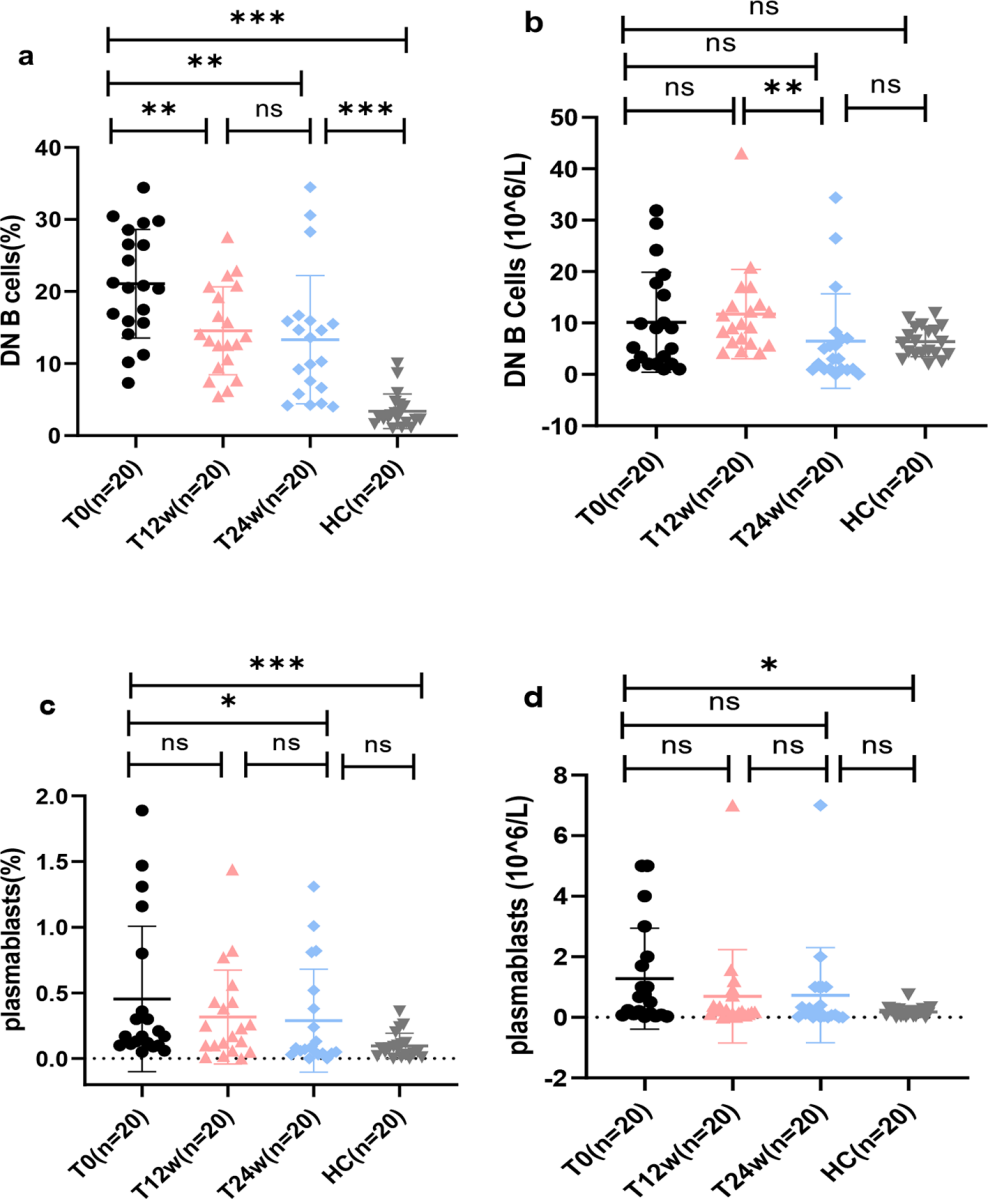
Effects of telitacicept combined with standard therapy on DN B cells and plasmablasts in patients with aPL-positive SLE. **(a)** DN B cells (%); **(b)** DN B cells (absolute counts); **(c)** plasmablasts (%); **(d)** plasmablasts (absolute counts). Data are presented at T0, T12w, T24w and healthy controls (HC). Abbreviations: DN B cells, double-negative B cells; HC, healthy controls. *p < 0.05; **p < 0.01; ***p < 0.001; ns, not significant.

**Figure 3:**
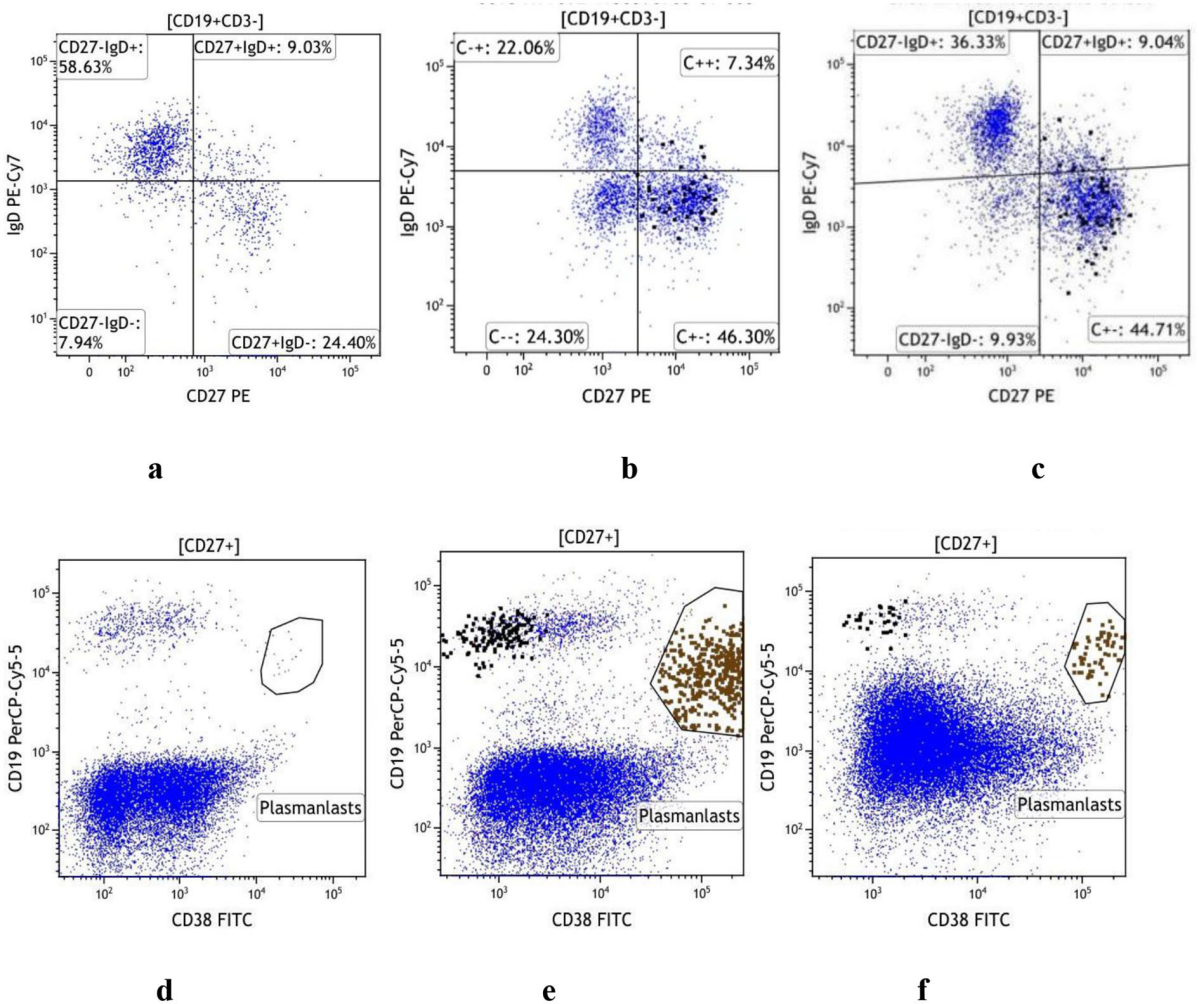
Flow cytometric analysis of DN B cells and circulating plasmablasts. **(a)** DN B cell expression in healthy participants. **(b)** Baseline DN B cell expression in a patient with aPL-positive SLE. **(c)** DN B cell expression at 24 weeks post-telitacicept treatment in the same patient. **(d)** plasmablasts expression in healthy participants. **(e)** Baseline plasmablasts expression in a patient with aPL-positive SLE. **(f)** plasmablasts expression at 24 weeks post-telitacicept treatment in the same patient.

Telitacicept combined with standard therapy induced significant alterations in B cell subsets in patients with aPL-positive SLE, particularly in circulating plasmablasts and DN B cells (**Figure 2**). As shown in **Figure 3**, representative flow cytometry plots of DN B cells and plasmablasts in PBMCs from a healthy control and two aPL-positive SLE patients before and at 24 weeks after telitacicept treatment are presented. At baseline, the proportions of DN B cells and plasmablasts among the total B cell population were notably higher than those in HCs (both *p <* 0.001) (**Figure 2a-c**). The absolute count of plasmablasts was also higher compared to that in HCs (*p <* 0.05) (**Figure 2d**). Although the absolute count of DN B cells did not differ significantly from that of the HCs, it exhibited an upward trend (**Figure 2b**).

At 12 weeks of treatment, the proportions of DN B cells had decreased significantly (*p* < 0.05) but did not change markedly between weeks 12 and 24. Although the levels remained higher than those in HCs at week 24, DN B cell proportions demonstrated an overall progressive decline throughout the treatment course (**Figure 2a**).

However, the absolute DN B cell counts did not differ significantly across the four groups, except for a significant decrease between T12W and T24W (p = 0.01). DN B cell numbers demonstrated a downward trend after 24 weeks of treatment, with levels approaching those observed in HCs (**Figure 2b**).

At the 12-week follow-up, neither the proportion nor the absolute count of plasmablasts differed significantly from the baseline, with no additional changes observed between weeks 12 and 24. However, by week 24, the proportion of plasmablasts had decreased significantly compared to the baseline (*p* < 0.05), reaching levels comparable to those of the HCs (**Figure 2c**). Although the absolute plasmablast count did not differ significantly, it exhibited a clear downward trend over the course of the telitacicept treatment (**Figure 2d**).

### 3.4 Correlation between peripheral blood lymphocyte subsets and clinical parameters in patients with aPL-positive SLE at baseline

**Figure 4:**
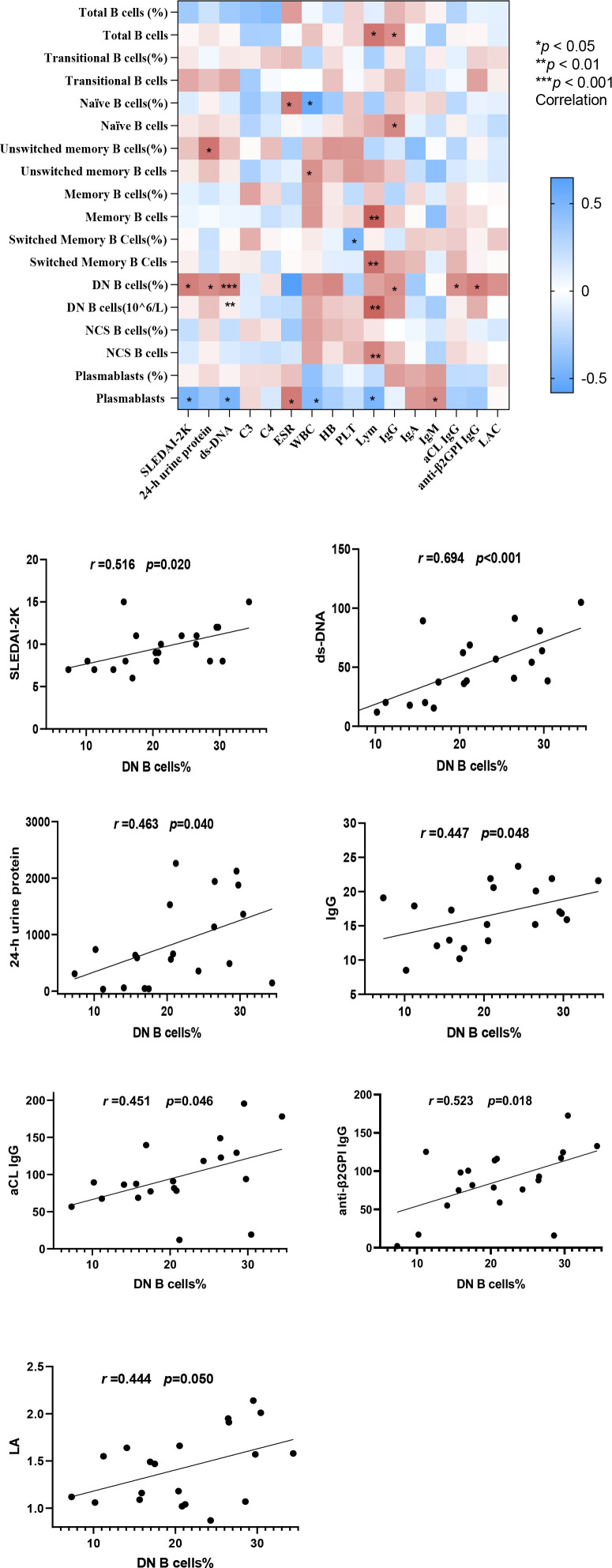
Correlation analysis between lymphocyte subsets and clinical parameters in patients with aPL-positive SLE.

We assessed the correlations between the proportions and counts of total B cells, transitional B cells, naïve B cells, unswitched memory B cells, memory B cells, switched memory B cells, DN B cells, unswitched B cells, and plasmablasts with various clinical measures in the 20 patients with aPL-positive SLE. The parameters included SLEDAI-2K, 24-h urine protein, ds-DNA, C3, C4, ESR, WBC count, lymphocyte count, and IgG, IgA, IgM, aCL IgG, anti-β2GPI IgG, and LAC levels. In patients with aPL-positive SLE, the proportion of DN B cells exhibited significant positive correlations with both disease activity and serological parameters, including SLEDAI-2K (r = 0.516, *p* = 0.020), 24-h urine protein (r = 0.463, *p* = 0.040), ds-DNA (r = 0.694, *p* < 0.001), serum IgG (r = 0.447, *p* = 0.048), aCL IgG (r = 0.451, *p* = 0.046), anti-β2GPI IgG (r = 0.523, *p* = 0.018), and LAC (r = 0.444, *p* = 0.050) (**Figure 4**).

### 3.5 Changes in cytokine levels and correlation between DN B cells and IL-21 in patients with aPL-positive SLE

**Figure 5:**
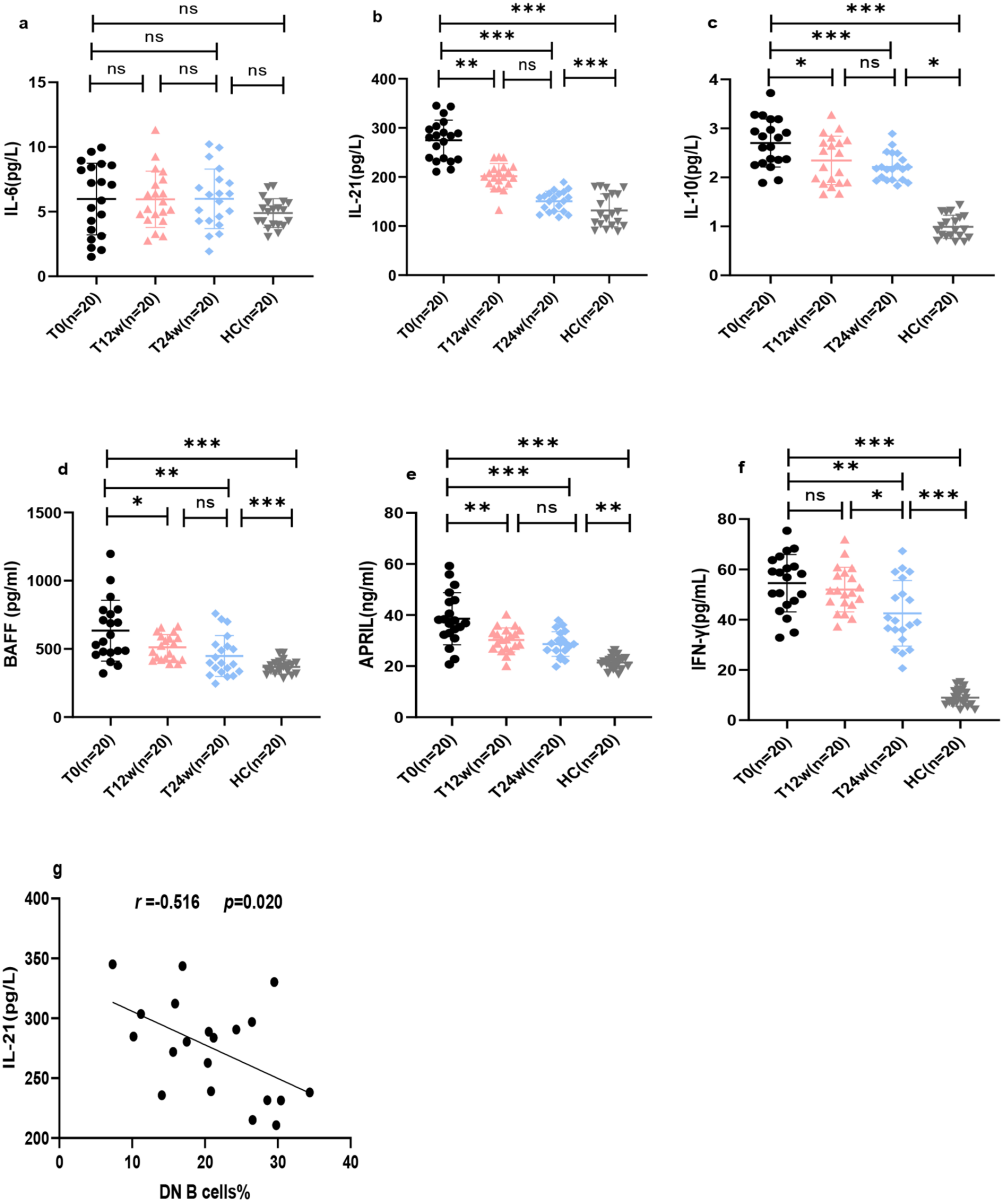
Changes in cytokines and correlation analysis following telitacicept treatment in patients with aPL-positive SLE. **(a)** IL-6 levels; **(b)** IL-21 levels; **(c)** IL-10 levels; **(d)** BAFF levels; **(e)** APRIL levels; **(f)** IFN-γ levels; **(g)** correlation between IL-21 levels and percentage of DN B cells. Data are presented for T0, T12w, T24w and HC. Correlation analysis was performed using Spearman’s correlation test. *p < 0.05; **p < 0.01; ***p < 0.001; ns, not significant.

To evaluate the relationship between DN B cells and inflammation in patients with aPL-Positive SLE, we measured IL-6, IL-21, IL-10, BAFF, APRIL and IFN-γ levels. We found that BAFF, APRIL, IFN-γ, IL-21, and IL-10 levels were significantly higher in the SLE group than in the HC group (*p* < 0.001) (**Figures 5b-f**). Notably, these elevated cytokine levels were significantly reduced after 24 weeks of telitacicept treatment (**Figures 5b-f**). However, no significant changes in IL-6 levels were observed before or after treatment (**Figure 5a**). Concurrently, we observed a negative correlation between serum IL-21 levels and DN B cells (*r* = -0.516, *p =* 0.020) (**Figure 5g**).

### 3.6 Baseline predictors of SRI-4 response at week 24 by univariate and multivariable logistic regression

At the 24-week mark, all patients were followed up; 13 of the 20 patients (65%) achieved an SRI-4 response, while the remaining seven (35%) did not. In the univariate analysis, higher baseline IgG levels (OR = 1.56, 95% CI 1.06–2.29, P = 0.025), BAFF levels (OR = 1.02, 95% CI 1.00–1.03, P = 0.044), DN B cells (%) (OR = 1.54, 95% CI 1.03–2.31, P = 0.035), and anti-dsDNA levels (OR = 1.05, 95% CI 1.00–1.11, P = 0.049) were associated with SRI-4 response. In addition, 24-h urine protein (OR = 1.00, 95% CI 1.00–1.01, P = 0.069) and anti-β2GPI IgG levels (OR = 1.03, 95% CI 1.00–1.06, P = 0.068) showed borderline associations. Variables with P < 0.10 in the univariate analysis were subsequently entered into a multivariable logistic regression model. In the multivariable analysis, higher baseline IgG levels (OR = 1.95, 95% CI 1.05–3.63, P = 0.035) and anti-β2GPI IgG levels (OR = 1.06, 95% CI 1.01–1.12, P = 0.048) remained independently associated with SRI-4 response (**Table 2**).

**Table 2:** Baseline predictors of SRI-4 response at week 24 by univariate and multivariable logistic regression.

Variable	Univariate OR (95% CI)	*p*	Multivariate OR (95% CI)	*p*
IgG levels (g/L)	1.56 (1.06**–**2.29)	0.025	1.95 (1.05**–**3.63)	**0.035**
Serum anti-dsDNA levels(IU/mL)	1.05 (1.00**–**1.11)	0.049	/	**/**
24-h urine protein (mg/24h)	1.00 (1.00**–**1.01)	0.069	/	**/**
Anti-β2GPI IgG levels (CU)	1.03 (1.00**–**1.06)	0.068	1.06 (1.01**–**1.12)	**0.048**
BAFF levels (pg/mL)	1.02 (1.00**–**1.03)	0.044	/	**/**
DN B cells (%)	1.54 (1.03**–**2.31)	0.035	/	/

Variables with P < 0.10 in univariate analysis were considered for inclusion in the multivariable logistic regression model. Given the limited number of events, the number of variables included in the multivariable model was restricted to minimize potential overfitting.

### 3.7 Safety profile of telitacicept in patients with aPL-positive SLE

**Table 3** summarizes the adverse events (AEs) documented during the observation period. Notably, no severe AEs were reported in any patient, and three AEs were recorded following the initiation of telitacicept. During telitacicept treatment, infections occurred in 10% of the patients, whereas injection site reactions were observed in 5%. During telitacicept treatment, no thrombotic events or non-criteria manifestations of APS were observed in APS patients or aPL carriers. As no pregnancies occurred, obstetric complications were not assessed in this study.

**Table 3:** Adverse events associated with telitacicept therapy.

Adverse events	N (%)
All adverse events after initiating therapy, n (%)	3 (15%)
Serious adverse events, n (%)	0 (0.0)
Injection site reactions, n (%)	1 (5%)
Infection, n (%)	2 (10%)
Upper respiratory tract infection, n (%)	1 (5%)
Herpes simplex, n (%)	1 (5%)

## 4 Discussion

SLE is a chronic autoimmune disease in which the immune system attacks healthy tissues and cells. The dyregulated production and elimination of antibodies, circulation and tissue deposition of immune complexes, and complement and cytokine activation lead to several clinical manifestations of the disease and a relapsing-remitting disease course (1, 2). As a highly heterogeneous autoimmune condition, SLE is characterized by marked immune dysregulation, the generation of pathogenic autoantibodies, and multiorgan damage driven by immune complex deposition and inflammation in affected tissues (22, 23). aPL, including aCL and anti-β2GPI, antibodies and LAC, are hallmark autoantibodies associated with APS, a distinct systemic autoimmune disorder characterized by recurrent arterial or venous thrombosis, pregnancy complications, and diverse nonthrombotic clinical features (24). These autoantibodies, particularly triple aPL positivity, persistently positive moderate-to-high titers of aCL, and LAC positivity, are linked to an increased risk of thrombosis and/or pregnancy morbidity (25). They may appear in primary APS or in association with other autoimmune diseases, most typically SLE (4). Furthermore, the coexistence of SLE in aPL-positive patients is associated with an increased risk of thrombosis (26). However, no clear or specific treatment guidelines exist for managing aPL-positive patients with SLE. Notably, a recent study reported that SLE secondary APS was treated with anticoagulant therapy alone or in combination with antiplatelet agents, often along with antimalarial drugs (27). However, safe and effective targeted therapies are lacking. Although the precise pathogenesis of SLE with aPL positivity remains unclear, aberrant innate immune responses are believed to contribute to tissue injury through the release of inflammatory cytokines and abnormal activation of autoreactive T and B cells in SLE (28). Moreover, other studies indicate that B cells play a crucial role in disease development via the production of autoantibodies and abnormal regulation of immune responses (22). B cells are increasingly being recognized as key contributors to APS pathogenesis, particularly through their role in aPL production (14). Therefore, we initially characterized and compared the peripheral blood B cell compartment between patients with aPL-positive SLE and HCs to elucidate changes in specific B cell subsets potentially involved in the pathogenesis of aPL-positive SLE. Our findings suggest that targeting B cells may represent an effective and selective immunosuppressive strategy in patients with aPL-positive SLE. Rituximab, a chimeric monoclonal antibody targeting anti-CD20 on B cells, has been used to treat APS and provided therapeutic benefits to patients with APS (12). However, the therapeutic efficacy of rituximab in APS is less robust than anticipated when compared to that in other autoimmune diseases (14). Since anti-CD20 therapy does not eliminate plasma cells, this limited efficacy may be attributed to the persistence of aPL-producing plasma cells (14). Consequently, available evidence does not support its use as a first-line treatment for catastrophic APS, and its use is mainly restricted to severe or refractory cases (29). Belimumab, a monoclonal antibody targeting BAFF (also known as B lymphocyte stimulator or BlyS), is another B cell-directed approach that is approved for the treatment of patients with SLE. Clinical studies have demonstrated that BAFF levels are elevated in patients with either primary or secondary APS (30, 31). Evidence from small-scale studies has shown that belimumab reduces aCL and anti-β2GPI IgG titers (32, 33). However, a *post hoc* analysis revealed that in patients with aPL-positive SLE IgA aCL levels decreased significantly, whereas IgG and IgM aCL levels exhibited little to no change following belimumab treatment. Therefore, current literature provides insufficient evidence to establish a clear effect of rituximab or belimumab on aPL profiles, and identifying the most efficacious therapy still represents a substantial clinical challenge.

B cell dysregulation plays a key role in both SLE pathogenesis and aPL production (5, 8). Therefore, targeting B cells holds particular significance for treating aPL-positive SLE. Current therapeutic strategies include direct B cell depletion, modulation of B cell survival, and inhibition of antigen receptor-mediated B cell function. BAFF is a ligand in the tumor necrosis factor cytokine family and is expressed as a membrane-bound or soluble protein. It is a crucial B cell survival factor with key roles in B cell maturation and antibody production and class switching (34–36). BAFF overexpression has been implicated in the pathogenesis of SLE (37). APRIL, another B cell-activating factor from the tumor necrosis factor family, mediates its effects through the cell surface receptor transmembrane activator, TACI, and B cell maturation antigen (BCMA) (38). Moreover, compared to BAFF, APRIL has a higher binding affinity with TACI and BCMA. The binding of BAFF or APRIL to their receptors promotes B cell differentiation and proliferation, immunoglobulin production, and the upregulation of B cell effector molecule expression (39, 40), which play vital roles in the pathogenesis of SLE (41). Telitacicept is a novel fusion protein consisting of a recombinant TACI receptor fused to the fragment crystallizable (Fc) domain of IgG. It was developed by Yantai Rongchang Pharmaceutical through its subsidiary RemeGen for the treatment of B cell-mediated autoimmune diseases and was first approved in China for the treatment of patients with active SLE (16). Its efficacy and safety have been demonstrated in a randomized, double-blind, phase 2b study involving patients with SLE (7). Telitacicept functions by binding to and inhibiting the activities of both BLyS and APRIL, thus suppressing the maturation and survival of plasma cells and fully developed B cells (15), and may hold considerable promise for effectively suppressing aPL synthesis. However, a critical knowledge gap remains in identifying patients with aPL-positive SLE that would derive maximal therapeutic benefit from using telitacicept. Therefore, it is crucial to identify predictive biomarkers to determine patients who are most likely to benefit from telitacicept, thereby facilitating the development of precision medicine and cost-effective therapies in clinical practice. In our study, we explored B cell subset dynamics over 6 months of telitacicept combined with standard therapy in aPL-positive SLE patients. However, the absolute DN B cell counts did not differ significantly across the four groups, consistent with previous reports in SLE (42). In contrast, the proportion of DN B cells gradually declined following telitacicept treatment, suggesting that BAFF/APRIL pathway blockade may inhibit the expansion of these potentially pathogenic B-cell populations. Plasmablast proportions decreased by week 24 and approached levels observed in healthy controls, indicating a reduction in antibody-secreting activity. Importantly, by focusing on aPL-positive SLE patients, a high-risk subgroup not previously studied, we observed changes in DN B cells and plasmablasts that may serve as potential indicators of treatment response, though validation in larger cohorts is required.

We observed marked decreases in SLEDAI-2K scores, serum IgG levels, ESR, and anti-dsDNA antibody titers, along with increased C3 and C4 complement levels following treatment with telitacicept, which is consistent with findings from previous studies (42). Notably, decreased aCL IgG, anti-β2GPI IgG, and LAC levels were also observed following treatment with telitacicept. To the best of our knowledge, this is the second retrospective cohort report to evaluate telitacicept therapy in patients with aPL-positive SLE, and our findings are consistent with those of the first retrospective cohort study investigating telitacicept use in this patient population (19). Notably, a pronounced rebound in aPL titers was observed in one patient following telitacicept withdrawal (19), which further supports its role in suppressing plasma cell-derived aPL antibody production.

Moreover, our findings revealed significant alterations in circulating B cell subsets among patients with aPL-positive SLE treated with telitacicept, particularly in circulating IgD^-^CD27^-^ DN B cells and plasmablasts. Importantly, this is the first observational cohort study documenting the dynamic changes in circulating B cell subsets following telitacicept therapy in patients with aPL-positive SLE. Furthermore, plasmablasts were elevated in this patient population compared to the HCs. Consistent with these observations, a previous study reported a high frequency of plasmablasts in patients with SLE, with these cells exhibiting a strong correlation with lupus disease activity and anti-dsDNA antibody titers (43). Notably, Hisada et al. confirmed elevated plasmablast levels in 26 patients with primary antiphospholipid syndrome (PAPS) and 19 patients with secondary APS associated with SLE (44). Wei et al. proposed that DN B cells represent a novel memory B cell subset and demonstrated their aberrant expansion in individuals with SLE (45). This finding has been supported by additional studies (46, 47), which have reported DN B cell expansion in patients with SLE. Notably, one study found that patients with lupus nephritis (LN) had a significantly higher percentage of DN B cells than those without LN, suggesting that DN B cells may play a pathogenic role in the renal damage associated with SLE. In our study, we similarly observed that the proportion of DN B cells among the total B cells at baseline was markedly higher in the patients than in HCs. Additionally, the number of DN B cells was positively correlated to SLEDAI-2K scores, ds-DNA titers, 24hPro, and serum IgG levels. Previous studies have demonstrated that peripheral DN B cells are significantly correlated with SLE disease activity, with patients with elevated DN B cell levels (reflecting greater disease activity) more frequently requiring treatment with glucocorticoids (46). Consistent with previous findings (47), DN B cell frequencies were positively correlated with 24hPro, suggesting that DN B cells may exhibit an increased tendency to differentiate into plasma cells that produce pathogenic autoreactive antibodies in SLE. Furthermore, our study also demonstrated that the proportion of DN B cells was positively correlated with both aCL IgG and anti-β2GPI IgG levels, with a particularly significant positive correlation observed for anti-β2GPI IgG. Telitacicept is well known to inhibit the terminal differentiation of mature B cells into plasma cells (48). Notably, a significant reduction in aCL IgG and anti-β2GPI IgG titers and LAC activity was observed following telitacicept treatment. Collectively, these results indicated that DN B cells readily differentiate into plasma cells that produce pathogenic aPL antibodies in patients with aPL-positive SLE.

Notably, we observed an increase in IL-21, IL-10, BAFF, APRIL and IFN-γ levels in the aPL-positive SLE group compared to the HC group. Following 24 weeks of telitacicept treatment, these elevated cytokines were significantly reduced. These findings suggest that dysregulation of the cytokine network implicated in B-cell activation and differentiation may contribute to the aberrant B-cell responses observed in aPL-positive systemic lupus erythematosus. IL-21 is a critical cytokine that regulates B cell differentiation. Elevated IL-21 levels promote autoantibody production in several autoimmune diseases, thereby playing a role in the development of pathological features (49). IL-21 potently induces B cell activation and promotes their differentiation into autoreactive plasma cells that secrete Ig in SLE (50). Consistent with previous findings (47), our findings revealed that serum IL-21 levels were significantly inversely correlated with the proportion of DN B cells. This is likely attributed to the depletion of IL-21 by DN B cells to facilitate their terminal differentiation into plasma cells, which are derived from plasmablasts. This finding may explain the increase in plasmablasts in patients with aPL-positive SLE prior to telitacicept treatment.

BAFF and APRIL are essential survival factors for B cells and play pivotal roles in maintaining autoreactive B-cell populations (36). Increased levels of these cytokines have been widely reported in SLE and are thought to support the persistence and expansion of pathogenic B-cell subsets (41). In this context, the observed reduction of BAFF and APRIL following telitacicept therapy likely contributes to the attenuation of aberrant B-cell activation and differentiation. In addition, we observed elevated levels of IFN-γ in patients with aPL-positive SLE, which were significantly reduced after treatment. IFN-γ has been widely recognized as an important pro-inflammatory cytokine involved in the immunopathogenesis of SLE and plays a critical role in promoting abnormal B-cell responses and autoantibody production (51). Mechanistically, IFN-γ can induce the production of BAFF by T cells (52) and antigen-presenting cells (53), thereby enhancing B-cell survival and activation. The decrease in IFN-γ levels after telitacicept therapy may reflect an improvement in the inflammatory environment associated with dysregulated B-cell activation. Notably, a reduction in DN B cells was also observed following treatment in our study. Although B-cell subsets in this study were analyzed at the level of DN B cells without further subdivision, emerging evidence suggests that double-negative 2 (DN2) B cells represent a pathogenic DN subset closely associated with IFN-γ signaling in SLE (54). Therefore, further studies with more detailed immunophenotyping are needed to determine whether DN2 B cells are preferentially affected by telitacicept therapy.

At the 24-week mark, all patients were followed up, and 65% achieved an SRI-4 response. Although previous studies have reported a low treatment response rate (42), our study enrolled a specific cohort of patients with aPL-positive SLE. In this study, we have explored baseline predictors of SRI-4 response at week 24 in patients with aPL-positive SLE treated with telitacicept. Multivariable logistic regression analysis demonstrated that higher baseline IgG levels and anti-β2GPI IgG levels were independently associated with SRI-4 response. These findings suggest that patients with higher baseline IgG and anti-β2GPI IgG levels, which may reflect increased B-cell–mediated immune activity, could be more likely to benefit from B-cell–targeted therapy. IgG levels represent the overall level of immunoglobulin production and B-cell activation in SLE. As a dual inhibitor of BAFF and APRIL, telitacicept inhibits B-cell survival and differentiation and subsequently reduces immunoglobulin production (16). Therefore, patients with higher baseline IgG levels may have more active humoral immune responses and may be more responsive to therapies targeting B-cell pathways. Interestingly, anti-β2GPI IgG levels were also independently associated with treatment response. Anti-β2GPI antibodies are key components of the antiphospholipid antibody profile and reflect autoreactive B-cell activity (14). Patients with higher baseline anti-β2GPI IgG levels may represent a subgroup with stronger B-cell–driven autoimmunity, which could partly explain the observed association with response to telitacicept. Taken together, these findings suggest that baseline immunological characteristics reflecting B-cell activation may help identify patients who are more likely to respond to B-cell–targeted therapies. Nevertheless, these findings should be interpreted with caution given the relatively small sample size and the limited number of non-responders in this exploratory study. Further large-scale studies are needed to validate these observations and to further define immunological predictors of treatment response in aPL-positive SLE. These biomarkers may serve as valuable tools for patient stratification and could facilitate the optimized clinical use of telitacicept in routine practice.

Furthermore, this study confirmed the safety of telitacicept in a subgroup of patients with aPL-positive SLE. Notably, no serious AEs were documented in any patient receiving telitacicept, which is consistent with the previously reported favorable safety profile of this drug in this population (19). Three mild to moderate AEs were recorded after telitacicept initiation, which included one injection site reaction and two infections, namely an upper respiratory tract infection and herpes simplex. The injection site reaction was mild in severity and did not require treatment interruption. Both infections developed after cold exposure; all symptoms resolved with symptomatic management, and telitacicept therapy was continued uninterrupted in all cases. Collectively, these findings demonstrate that telitacicept exhibited a favorable safety profile in this patient cohort.

Despite the promising findings of this study, several limitations should be acknowledged. First, this was a single-center study with a relatively small patient cohort and lacked an aPL-negative SLE control group. The lack of a control group limited our ability to distinguish the specific effects of telitacicept from those of concomitant therapies. In addition, the relatively small sample size restricted the number of variables that could be included in the multivariable logistic regression model in order to minimize the risk of model overfitting. Therefore, future studies with larger sample sizes are warranted to validate these findings. Furthermore, the patients received variable conventional therapeutic regimens during the study period, which may have influenced the observed differences in B cell subset frequencies. Moreover, extended follow-up may offer deeper insights into the factors influencing treatment outcomes in patients with aPL positivity. Finally, our flow cytometry panel was not designed to specifically identify atypical DN2 cells. DN2 cells have recently been shown to represent a distinct subset of IgD^-^CD27^-^ B cells with CXCR5^-^, CD11c^+^, and extra-follicular features, which are expanded in active SLE and contribute to pathogenic responses, including autoantibody production and interactions with granzyme K^+^ CD8 T cells in lupus kidneys (55, 56). Consequently, while our findings provide insight into B cell dynamics in SLE, they should be interpreted in the context of these atypical B cell subsets. Future studies incorporating markers specific for DN2 cells are warranted to clarify their contribution to SLE pathogenesis and to better understand the interplay between extrafollicular B cell responses and tissue inflammation.

In conclusion, our study provides novel insights suggesting that telitacicept combined with standard therapy may confer therapeutic benefits in patients with aPL-positive SLE. Treatment was associated with significant alterations in B-cell subsets, particularly DN B cells and plasmablasts, which may contribute to its therapeutic effects. Elevated baseline IgG and anti-β2GPI IgG levels may predict a favorable response to telitacicept and help identify patients most likely to benefit from this therapy. Further large-scale double-blind randomized trials are required to confirm these results.

## Data Availability

The raw data supporting the conclusions of this article will be made available by the authors, without undue reservation.

## References

[1] Elia A Zucchi D Silvagni E Oliva M Cascarano G Cardelli C . Systemic lupus erythematosus: one year in review 2025. Clin Exp Rheumatol. (2025) 43:397–406. doi: 10.55563/clinexprheumatol/m0pi1k. PMID: 40072872

[2] Kiriakidou M Ching C . Systemic lupus erythematosus. Ann Intern Med. (2020) 172:ITC81–96. doi: 10.7326/aitc202006020. PMID: 32479157

[3] Barbhaiya M Zuily S Naden R Hendry A Manneville F Amigo MC . The 2023 ACR/EULAR antiphospholipid syndrome classification criteria. Arthritis Rheumatol. (2023) 75:1687–702. doi: 10.1002/art.42624. PMID: 37635643

[4] Miyakis S Lockshin M Atsumi T Branch D Brey R Cervera R . International consensus statement on an update of the classification criteria for definite antiphospholipid syndrome (APS). J Thromb Haemost. (2006) 4:295–306. doi: 10.1111/j.1538-7836.2006.01753.x. PMID: 16420554

[5] Ji L Geng Y Zhang X Deng X Song Z Tan M . B cell pathway dual inhibition for systemic lupus erythematosus: a prospective single‐arm cohort study of telitacicept. MedComm. (2024) 5:e515. doi: 10.1002/mco2.515. PMID: 38525109 PMC10960726

[6] Krustev E Clarke A Barber M . B cell depletion and inhibition in systemic lupus erythematosus. Expert Rev Clin Immunol. (2023) 19:55–70. doi: 10.1080/1744666x.2023.2145281. PMID: 36342225

[7] Wu D Li J Xu D Merrill JT van Vollenhoven RF Liu Y . Telitacicept in patients with active systemic lupus erythematosus: results of a phase 2b, randomised, double-blind, placebo-controlled trial. Ann Rheum Dis. (2024) 83:475–87. doi: 10.1136/ard-2023-224854. PMID: 38129117 PMC10958275

[8] Tipton CM Hom JR Fucile CF Rosenberg AF Sanz I . Understanding B‐cell activation and autoantibody repertoire selection in systemic lupus erythematosus: a B‐cell immunomics approach. Immunol Rev. (2018) 284:120–31. doi: 10.1111/imr.12660. PMID: 29944759 PMC6022284

[9] Ruiz-Irastorza G Bertsias G . Treating systemic lupus erythematosus in the 21st century: new drugs and new perspectives on old drugs. Rheumatol (Oxford). (2020) 59:v69–81. doi: 10.1093/rheumatology/keaa403. PMID: 33280011 PMC7719039

[10] Carter LM Ehrenstein MR Vital EM . Evolution and trajectory of B-cell targeted therapies in rheumatic diseases. Lancet Rheumatol. (2025) 7:e355–67. doi: 10.1016/S2665-9913(24)00338-2. PMID: 40058377

[11] Chen L Luo X Yang Y Du X Zhang X Luo D . B cell-targeted therapies in systemic lupus erythematosus: current status and perspectives. Biochem Pharmacol. (2025) 239:117018. doi: 10.1016/j.bcp.2025.117018. PMID: 40473226

[12] Khattri S Zandman-Goddard G Peeva E . B-cell directed therapies in antiphospholipid antibody syndrome--new directions based on murine and human data. Autoimmun Rev. (2012) 11:717–22. doi: 10.1016/j.autrev.2011.12.011. PMID: 22269862

[13] Bayer G Agier M Lioger B Lepelley M Zenut M Lanoue M . Rituximab-induced serum sickness is more frequent in autoimmune diseases as compared to hematological Malignancies: a French nationwide study. Eur J Intern Med. (2019) 67:59–64. doi: 10.1016/j.ejim.2019.06.009. PMID: 31279430

[14] Dieudonné Y Guffroy A Poindron V Soulas Sprauel P Martin T Korganow A-S . B cells in primary antiphospholipid syndrome: review and remaining challenges. Autoimmun Rev. (2021) 20:102798. doi: 10.1016/j.autrev.2021.102798. PMID: 33722752

[15] Zeng L Yang K Wu Y Yu G Yan Y Hao M . Telitacicept: a novel horizon in targeting autoimmunity and rheumatic diseases. J Autoimmun. (2024) 148. doi: 10.1016/j.jaut.2024.103291. PMID: 39146891

[16] Dhillon S . Telitacicept: first approval. Drugs. (2021) 81:1671–5. doi: 10.1007/s40265-021-01591-1. PMID: 34463932

[17] Regola F Piantoni S Lowin T Archetti S Reggia R Kumar R . Association between changes in BLyS levels and the composition of B and T cell compartments in patients with refractory systemic lupus erythematosus treated with belimumab. Front Pharmacol. (2019) 10:433. doi: 10.3389/fphar.2019.00433. PMID: 31105569 PMC6494924

[18] Jin H Li Y Wang X Li Z Ma B Niu L . Efficacy and safety of telitacicept in patients with systemic lupus erythematosus: a multicentre, retrospective, real-world study. Lupus Sci Med. (2023) 10. doi: 10.1136/lupus-2023-001074. PMID: 38007228 PMC10679987

[19] Zhou L You Y Cai S Ye C Dong L . Effects of telitacicept in SLE patients with antiphospholipid antibody positivity: a retrospective self-controlled case series. Clin Rheumatol. (2025) 44:2287–97. doi: 10.1007/s10067-025-07411-1. PMID: 40317459

[20] Hochberg MC . Updating the American College of Rheumatology revised criteria for the classification of systemic lupus erythematosus. Arthritis Rheum. (1997) 40:1725. doi: 10.1002/art.1780400928. PMID: 9324032

[21] Furie RA Petri MA Wallace DJ Ginzler EM Merrill JT Stohl W . Novel evidence-based systemic lupus erythematosus responder index. Arthritis Rheum. (2009) 61:1143–51. doi: 10.1002/art.24698. PMID: 19714615 PMC2748175

[22] Karrar S Cunninghame Graham DS . Review: abnormal B cell development in systemic lupus erythematosus: what the genetics tell us. Arthritis Rheumatol. (2018) 70:496–507. doi: 10.1002/art.40396. PMID: 29207444 PMC5900717

[23] Liossis SN Staveri C . What's new in the treatment of systemic lupus erythematosus. Front Med (Lausanne). (2021) 8:655100. doi: 10.3389/fmed.2021.655100. PMID: 33748165 PMC7973110

[24] Tektonidou MG Andreoli L Limper M Amoura Z Cervera R Costedoat-Chalumeau N . EULAR recommendations for the management of antiphospholipid syndrome in adults. Ann Rheum Dis. (2019) 78:1296–304. doi: 10.1136/annrheumdis-2019-215213. PMID: 31092409 PMC11034817

[25] Pengo V Ruffatti A Legnani C Gresele P Barcellona D Erba N . Clinical course of high-risk patients diagnosed with antiphospholipid syndrome. J Thromb Haemost. (2010) 8:237–42. doi: 10.1111/j.1538-7836.2009.03674.x. PMID: 19874470

[26] Erkan D Harrison MJ Levy R Peterson M Petri M Sammaritano L . Aspirin for primary thrombosis prevention in the antiphospholipid syndrome: a randomized, double-blind, placebo-controlled trial in asymptomatic antiphospholipid antibody-positive individuals. Arthritis Rheum. (2007) 56:2382–91. doi: 10.1002/art.22663. PMID: 17599766

[27] Nagy N Papp G Gáspár-Kiss E Diószegi Á Tarr T . Changes in clinical manifestations and course of systemic lupus erythematosus and secondary antiphospholipid syndrome over three decades. Biomedicines. (2023) 11:1218. doi: 10.3390/biomedicines11041218. PMID: 37189836 PMC10136176

[28] Pan L Lu M Wang J Xu M Yang S . Immunological pathogenesis and treatment of systemic lupus erythematosus. World J Pediatr. (2020) 16:19–30. doi: 10.1007/s12519-019-00229-3. PMID: 30796732 PMC7040062

[29] Uthman I Noureldine MHA Ruiz-Irastorza G Khamashta MA . Management of antiphospholipid syndrome. Ann Rheum Dis. (2019) 78:155–61. doi: 10.1136/annrheumdis-2018-213846. PMID: 30282668

[30] Alvarez-Rodriguez L Riancho-Zarrabeitia L Calvo-Alén J López-Hoyos M Martínez-Taboada V . Peripheral B-cell subset distribution in primary antiphospholipid syndrome. Int J Mol Sci. (2018) 19:589. doi: 10.3390/ijms19020589. PMID: 29462939 PMC5855811

[31] van den Hoogen LL Palla G Bekker CPJ Fritsch-Stork RDE Radstake TRDJ van Roon JAG . Increased B-cell activating factor (BAFF)/B-lymphocyte stimulator (BLyS) in primary antiphospholipid syndrome is associated with higher adjusted global antiphospholipid syndrome scores. RMD Open. (2018) 4:e000693. doi: 10.1136/rmdopen-2018-000693. PMID: 30018806 PMC6045704

[32] Dong J Zhao L Pan L Wang H Wang L . Belimumab therapy for refractory immune thrombocytopenia in systemic lupus erythematosus patients with anti-phospholipid antibodies. Scand J Rheumatol. (2024) 53:59–62. doi: 10.1080/03009742.2023.2247881. PMID: 37650252

[33] Emmi G Bettiol A Palterer B Silvestri E Vitiello G Parronchi P . Belimumab reduces antiphospholipid antibodies in SLE patients independently of hydroxychloroquine treatment. Autoimmun Rev. (2019) 18:312–4. doi: 10.1016/j.autrev.2018.11.002. PMID: 30639638

[34] Castigli E Wilson SA Scott S Dedeoglu F Xu S Lam K-P . TACI and BAFF-R mediate isotype switching in B cells. J Exp Med. (2005) 201:35–9. doi: 10.1084/jem.20032000. PMID: 15630136 PMC2212754

[35] Batten M Groom J Cachero TG Qian F Schneider P Tschopp J . BAFF mediates survival of peripheral immature B lymphocytes. J Exp Med. (2000) 192:1453–66. doi: 10.1084/jem.192.10.1453. PMID: 11085747 PMC2193190

[36] Mackay F Browning JL . BAFF: a fundamental survival factor for B cells. Nat Rev Immunol. (2002) 2:465–75. doi: 10.1038/nri844. PMID: 12094221

[37] Steri M Orrù V Idda ML Pitzalis M Pala M Zara I . Overexpression of the cytokine BAFF and autoimmunity risk. N Engl J Med. (2017) 376:1615–26. doi: 10.1056/NEJMoa1610528. PMID: 28445677 PMC5605835

[38] Sakai J Akkoyunlu M . The role of BAFF system molecules in host response to pathogens. Clin Microbiol Rev. (2017) 30:991–1014. doi: 10.1128/cmr.00046-17. PMID: 28855265 PMC5608883

[39] Cancro MP D'Cruz DP Khamashta MA . The role of B lymphocyte stimulator (BLyS) in systemic lupus erythematosus. J Clin Invest. (2009) 119:1066–73. doi: 10.1172/jci38010. PMID: 19411764 PMC2673851

[40] Shabgah AG Shariati-Sarabi Z Tavakkol-Afshari J Mohammadi M . The role of BAFF and APRIL in rheumatoid arthritis. J Cell Physiol. (2019) 234:17050–63. doi: 10.1002/jcp.28445. PMID: 30941763

[41] Samy E Wax S Huard B Hess H Schneider P . Targeting BAFF and APRIL in systemic lupus erythematosus and other antibody-associated diseases. Int Rev Immunol. (2017) 36:3–19. doi: 10.1080/08830185.2016.1276903. PMID: 28215100

[42] Chen X Hu L Zhu L Tu J Gui J Fang M . Prospective analysis of B cell subset dynamics following telitacicept treatment in systemic lupus erythematosus. Arthritis Res Ther. (2025) 27:126. doi: 10.1186/s13075-025-03584-x. PMID: 40533865 PMC12175369

[43] Jacobi AM Mei H Hoyer BF Mumtaz IM Thiele K Radbruch A . HLA-DRhigh/CD27high plasmablasts indicate active disease in patients with systemic lupus erythematosus. Ann Rheum Dis. (2010) 69:305–8. doi: 10.1136/ard.2008.096495. PMID: 19196727

[44] Hisada R Kato M Sugawara E Kanda M Fujieda Y Oku K . Circulating plasmablasts contribute to antiphospholipid antibody production, associated with type I interferon upregulation. J Thromb Haemost. (2019) 17:1134–43. doi: 10.1111/jth.14427. PMID: 30864219

[45] Wei C Anolik J Cappione A Zheng B Pugh-Bernard A Brooks J . A new population of cells lacking expression of CD27 represents a notable component of the B cell memory compartment in systemic lupus erythematosus. J Immunol. (2007) 178:6624–33. doi: 10.4049/jimmunol.178.10.6624. PMID: 17475894

[46] Jenks SA Cashman KS Zumaquero E Marigorta UM Patel AV Wang X . Distinct effector B cells induced by unregulated Toll-like receptor 7 contribute to pathogenic responses in systemic lupus erythematosus. Immunity. (2018) 49:725–39. doi: 10.1016/j.immuni.2018.08.015. PMID: 30314758 PMC6217820

[47] You X Zhang R Shao M He J Chen J Liu J . Double negative B cell is associated with renal impairment in systemic lupus erythematosus and acts as a marker for nephritis remission. Front Med (Lausanne). (2020) 7:85. doi: 10.3389/fmed.2020.00085. PMID: 32318574 PMC7155774

[48] Cheng J Peng Y Wu Q Wu Q He J Yuan G . Efficacy and safety of telitacicept therapy in systemic lupus erythematosus with hematological involvement. Clin Rheumatol. (2024) 43:2229–36. doi: 10.1007/s10067-024-06992-7. PMID: 38767710

[49] Ettinger R Kuchen S Lipsky PE . The role of IL-21 in regulating B-cell function in health and disease. Immunol Rev. (2008) 223:60–86. doi: 10.1111/j.1600-065X.2008.00631.x. PMID: 18613830

[50] Wang S Wang J Kumar V Karnell JL Naiman B Gross PS . IL-21 drives expansion and plasma cell differentiation of autoreactive CD11chiT-bet+ B cells in SLE. Nat Commun. (2018) 9. doi: 10.1038/s41467-018-03750-7. PMID: 29717110 PMC5931508

[51] Liu W Zhang S Wang J . IFN-gamma, should not be ignored in SLE. Front Immunol. (2022) 13:954706. doi: 10.3389/fimmu.2022.954706. PMID: 36032079 PMC9399831

[52] Harigai M Kawamoto M Hara M Kubota T Kamatani N Miyasaka N . Excessive production of IFN-gamma in patients with systemic lupus erythematosus and its contribution to induction of b lymphocyte stimulator/B cell-activating factor/TNF ligand superfamily-13B. J Immunol. (2008) 181:2211–9. doi: 10.4049/jimmunol.181.3.2211. PMID: 18641361

[53] Scapini P Carletto A Nardelli B Calzetti F Roschke V Merigo F . Proinflammatory mediators elicit secretion of the intracellular B-lymphocyte stimulator pool (BLyS) that is stored in activated neutrophils: implications for inflammatory diseases. Blood. (2005) 105:830–7. doi: 10.1182/blood-2004-02-0564. PMID: 15358625

[54] Zumaquero E Stone SL Scharer CD Jenks SA Nellore A Mousseau B . IFNgamma induces epigenetic programming of human T-bet(hi) B cells and promotes TLR7/8 and IL-21 induced differentiation. Elife. (2019) 8. doi: 10.7554/eLife.41641. PMID: 31090539 PMC6544433

[55] Wu C Jiang S Chen Z Li T Gu X Dai M . Single-cell transcriptomics reveal potent extrafollicular B cell response linked with granzyme K(+) CD8 T cell activation in lupus kidney. Ann Rheum Dis. (2024) 84(3):451–66. doi: 10.1136/ard-2024-225876. PMID: 39419536

[56] Jenks SA Cashman KS Zumaquero E Marigorta UM Patel AV Wang X . Distinct effector B cells induced by unregulated Toll-like receptor 7 contribute to pathogenic responses in systemic lupus erythematosus. Immunity. (2018) 49:725–39:e726. doi: 10.1016/j.immuni.2018.08.015. PMID: 30314758 PMC6217820

